# Rozanolixizumab in generalized myasthenia gravis: Pooled analysis of the Phase 3 MycarinG study and two open-label extensions

**DOI:** 10.1177/22143602241305511

**Published:** 2025-03-04

**Authors:** Vera Bril, Artur Drużdż, Julian Grosskreutz, Ali A Habib, Renato Mantegazza, Sabrina Sacconi, Kimiaki Utsugisawa, Tuan Vu, Marion Boehnlein, Bernhard Greve, Maryam Gayfieva, Franz Woltering, Thaïs Tarancón, John Vissing, on behalf of the MycarinG, MG0004 and MG0007 study investigators

**Affiliations:** 1 University Health Network, Toronto, ON, Canada; 2 Department of Neurology, Municipal Hospital Poznań, Poznań, Poland; 3 Precision Neurology, Department of Neurology, University of Lübeck, Lübeck, Germany; 4 MDA ALS & Neuromuscular Center, Department of Neurology, University of California, Irvine, Orange, CA, USA; 5 Emeritus and Past Director, Department of Neuroimmunology and Neuromuscular Diseases, Fondazione Istituto di Ricovero e Cura a Carattere Scientifico, Istituto Nazionale Neurologico Carlo Besta, Milan, Italy; 6 Université Côte d’Azur, Peripheral Nervous System and Muscle Department, Pasteur 2 Hospital, Centre Hospitalier Universitaire de Nice, Nice, France; 7 Department of Neurology, Hanamaki General Hospital, Hanamaki, Iwate Prefecture, Japan; 8 Department of Neurology, University of South Florida Morsani College of Medicine, Tampa, FL, USA; 9 UCB, Monheim, Germany; 10 UCB, Slough, UK; 11 UCB, Madrid, Spain; 12 Department of Neurology, Rigshospitalet, University of Copenhagen, Copenhagen, Denmark

**Keywords:** ACh receptor, clinical efficacy, Fc receptor, monoclonal antibody, MuSK MG, myasthenia gravis, myasthenia gravis, generalized

## Abstract

**Background:**

Myasthenia gravis (MG) is a chronic autoimmune disease causing fluctuating muscle weakness. The MycarinG study showed that rozanolixizumab, a neonatal Fc receptor inhibitor, provided clinically meaningful improvements in MG outcomes in patients with acetylcholine receptor (AChR) and muscle-specific tyrosine kinase (MuSK) autoantibody-positive generalized MG (gMG).

**Objective:**

We assessed efficacy and safety of 6-week rozanolixizumab treatment cycles in patients with gMG.

**Methods:**

Following MycarinG, eligible patients enrolled in the open-label extension Phase 3 studies MG0004 (NCT04124965) to receive up to 52 weekly rozanolixizumab infusions or MG0007 (NCT04650854) to receive cycles of 6 weekly rozanolixizumab infusions (initiated on symptom worsening at investigators’ discretion). To assess the effect of repeated cyclical treatment, data were pooled across MycarinG, MG0004 (first 6 weeks) and MG0007 (interim analysis). Efficacy endpoints included change from baseline in Myasthenia Gravis Activities of Daily Living (MG-ADL), Myasthenia Gravis Composite (MGC) and Quantitative Myasthenia Gravis (QMG) assessed in patients who received ≥2 symptom-driven treatment cycles. Treatment-emergent adverse events (TEAEs) were assessed in patients who received ≥1 cycle and had an (up to) 8-week follow-up period.

**Results:**

At data cut-off (July 8, 2022), 188/196 (95.9%) patients received ≥1 treatment cycle with a follow-up period (primary safety pool; MycarinG/MG0007) and 127 (64.8%) received ≥2 symptom-driven cycles (primary efficacy pool; MycarinG/MG0004 [first 6 weeks]/MG0007). Consistent and clinically meaningful improvements in MG-ADL, MGC and QMG scores, and high MG-ADL, MGC and QMG response rates, were observed at the end of the first symptom-driven cycle and subsequent cycles. TEAEs were experienced by 169/188 (89.9%) patients and were mostly mild to moderate. TEAEs did not increase with repeated cycles.

**Conclusions:**

Repeated cycles of rozanolixizumab resulted in consistent, clinically meaningful improvements across cycles in MG-specific outcomes with an acceptable safety profile, supporting rozanolixizumab as a treatment option for adults with AChR and MuSK autoantibody-positive gMG.

## Plain language summary

The Phase 3 MycarinG study showed that weekly rozanolixizumab treatment for 6 weeks effectively reduced the severity of generalized myasthenia gravis (gMG) symptoms compared with placebo. After MycarinG, two additional studies (MG0004 and MG0007) were started to confirm whether rozanolixizumab continues to be effective and safe over long-term treatment of MG.

In MG0004, patients were treated with rozanolixizumab every week for up to 52 weeks. After MG0004 was started, MG0007 was set up to assess repeated 6-week treatment cycles of rozanolixizumab, with more cycles given only if a patient's symptoms worsened after they finished a rozanolixizumab treatment cycle. Once MG0007 was open, patients could move from MG0004 to MG0007 or enter MG0007 directly from MycarinG. This analysis includes data from repeated 6-week treatment cycles of patients across MycarinG, MG0004 and MG0007.

Repeated 6-week treatment cycles of rozanolixizumab in 127 patients over approximately a year demonstrated a consistent improvement in the severity of MG symptoms across multiple treatment cycles according to multiple measurements. Rozanolixizumab was effective in patients with both acetylcholine receptor and muscle-specific tyrosine kinase autoantibody-positive gMG. The most common time between cycles was between 4 –<8 weeks. In the 188 patients analyzed for safety, 169 (89.9%) experienced an adverse event. Repeated 6-week treatment cycles with rozanolixizumab over the long term effectively reduced MG symptoms, with adverse events that were mostly mild to moderate in severity and did not increase in frequency or severity with repeated rozanolixizumab treatment cycles.

## Introduction

Myasthenia gravis (MG) is a rare, chronic autoimmune disease characterized by fluctuating muscle weakness that has a significant impact on patients’ lives and may cause life-threatening crises.^1–3^ In acetylcholine receptor autoantibody-positive generalized myasthenia gravis (AChR Ab+ gMG), immunoglobulin G (IgG) subtypes 1 and 3 autoantibodies target AChRs, causing damage to the post-synaptic membrane of the neuromuscular junction via complement activation, functional blockade of AChR interaction with acetylcholine and receptor modulation.^1,4^ In muscle-specific tyrosine kinase autoantibody-positive (MuSK Ab+) gMG, IgG4 autoantibodies bind MuSK, reducing post-synaptic AChR clustering, resulting in impaired neuromuscular transmission.^1^ MuSK Ab+ gMG can be particularly severe and is more difficult to treat than AChR Ab+ gMG, with some treatments, such as complement inhibitors, unsuitable for use in this group of patients.^5,6^

Some patients have inadequately controlled symptoms despite receiving conventional treatments.^7,8^ Corticosteroids and non-steroidal immunosuppressant therapy are associated with short- and long-term adverse effects, such as increased risk of infection, diabetes, osteoporosis and hypertension.^1,9,10^ Plasma exchange (PLEX) and intravenous immunoglobulin (IVIg) are lengthy treatments often administered over several days.^10,11^ Furthermore, PLEX is an invasive treatment and the supply of IVIg is inconsistent, which can limit its use in MG treatment.^12^

One therapeutic target is the neonatal Fc receptor (FcRn), which provides a salvage and recycling mechanism that prolongs the half-life of IgG in serum, including pathogenic IgG1–4 autoantibodies, by preventing degradation in lysosomes.^13^ Rozanolixizumab is a humanized monoclonal antibody (mAb) that blocks the IgG binding region of FcRn. By inhibiting IgG salvage and recycling, IgG destruction is accelerated, thereby reducing serum IgG levels, including pathogenic IgG autoantibodies.^13^

MycarinG (NCT03971422; MG0003) was a Phase 3 study of a single cycle of six weekly doses of rozanolixizumab 7 mg/kg and 10 mg/kg.^14^ Rozanolixizumab demonstrated clinically meaningful and statistically significant differences from placebo with multiple MG-specific endpoints, and both doses were well tolerated. Additional data were needed to understand the safety and efficacy of rozanolixizumab with repeated treatment cycles. Following MycarinG, two open-label extension (OLE) Phase 3 studies were initiated: MG0004 (NCT04124965) was an OLE study of chronic weekly treatment of rozanolixizumab 7 mg/kg and 10 mg/kg for up to 52 weeks; MG0007 (NCT04650854) is an ongoing OLE study of 6-week cycles of weekly rozanolixizumab 7 mg/kg and 10 mg/kg. In this analysis, the efficacy and safety of repeated rozanolixizumab cycles in patients with gMG is assessed.

## Materials and methods

### MycarinG

MycarinG was a randomized, double-blind, placebo-controlled study, in which adults with AChR or MuSK Ab+ gMG (Myasthenia Gravis Foundation of America [MGFA] class II–IVa, Myasthenia Gravis Activities of Daily Living [MG-ADL] score of ≥3 [with ≥3 points for non-ocular symptoms] and a Quantitative Myasthenia Gravis [QMG] score of ≥11) were enrolled.^14^ Patients were randomized 1:1:1 to receive weekly doses of subcutaneous rozanolixizumab 7 mg/kg or 10 mg/kg or placebo for 6 weeks, followed by an 8-week observation period. Full methodology and results for MycarinG have been previously published.^14^

### OLE studies (MG0004 and MG0007)

**Figure 1.**
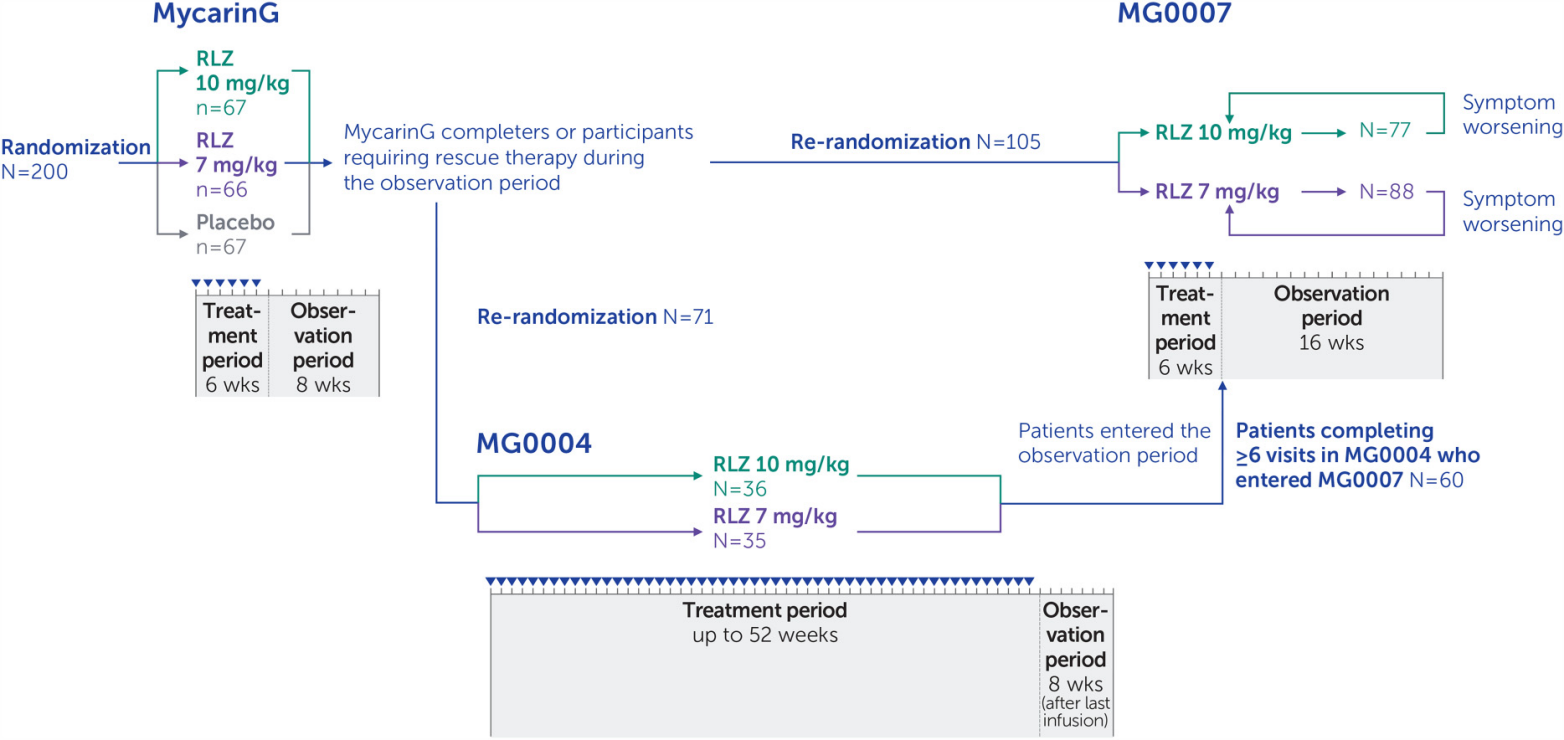
Pooled analysis study design.

Patients who completed the observation period of MycarinG, or required (but chose not to receive) rescue therapy due to disease worsening during the observation period of MycarinG, were eligible to enroll into MG0004 (if MG0007 was not yet open at their site) or MG0007 (Figure 1; Supplementary methods).

All patients. Treatment group assignments are based on randomization in MycarinG, the first dose received in MG0004, and highest dose received in the initial cycle of MG0007. MG, myasthenia gravis; MG-ADL, Myasthenia Gravis Activities of Daily Living; OLE, open-label extension; QMG, Quantitative Myasthenia Gravis; RLZ, rozanolixizumab.

When entering MG0004 from MycarinG, patients were re-randomized 1:1 to (up to 52) weekly subcutaneous infusions of open-label rozanolixizumab 7 mg/kg or 10 mg/kg, followed by an observation period of 8 weeks, with treatment administered by a healthcare professional at the clinical site or at home. In MG0004, if a patient experienced disease worsening they could receive rescue therapy (IVIg or PLEX) at the investigator's discretion, in which case rozanolixizumab treatment was discontinued or paused for a minimum of 2 weeks. MG0004 was closed early in response to feedback from clinicians and patients regarding the burden of the study, particularly the requirement for patients to attend weekly visits to the study center for up to 52 weeks of treatment administration. Furthermore, chronic weekly dosing was not anticipated to be used in clinical practice.

**Figure 2.**
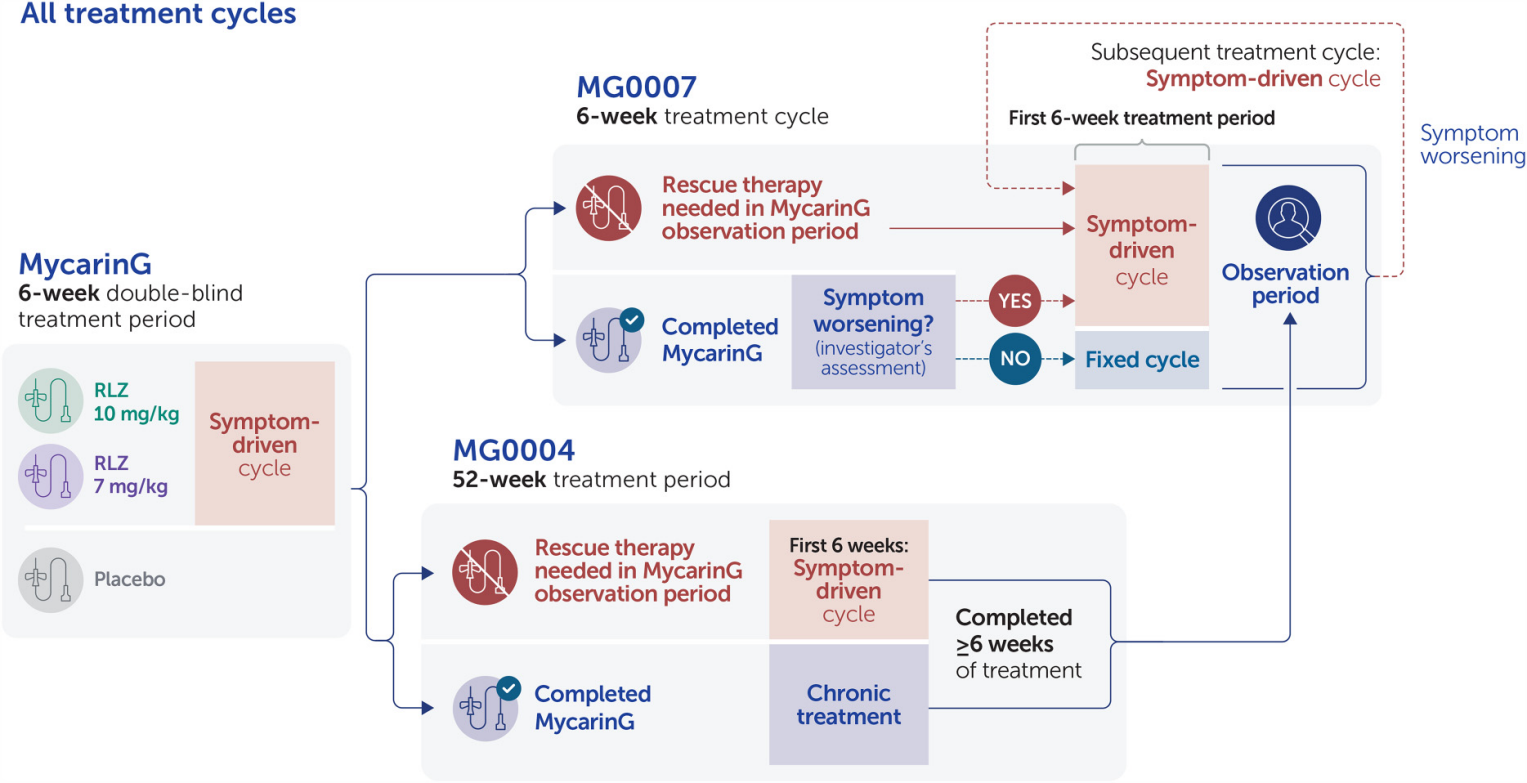
Classification of symptom-driven rozanolixizumab treatment cycles in the efficacy pools.

In MG0007, to tailor treatment to individual patients’ needs and to better reflect its expected use in the clinical setting, patients received cycles of 6 weekly rozanolixizumab infusions, initiated based on symptom worsening. Once MG0007 was initiated at the clinical site, eligible patients from MycarinG moved directly into MG0007; these patients were randomized to receive an initial 6-week cycle of subcutaneous rozanolixizumab 7 mg/kg or 10 mg/kg. Treatment cycles were followed by an observation period of up to 16 weeks; thereafter, patients continued to be monitored. After the initial cycle in MG0007, if a patient's symptoms worsened at any point during the observation period or afterwards, at the investigator's discretion, the patient received a further 6-week cycle of rozanolixizumab, referred to as a symptom-driven cycle (Figure 2). Advisory protocol guidance on symptom worsening for investigators to consider a new cycle at their discretion was, for example, when MG-ADL scores worsened by ≥2 points or QMG scores worsened by ≥3 points.

RLZ, rozanolixizumab.

In MycarinG, the 6 weeks of rozanolixizumab treatment was considered a symptom-driven cycle. For patients who needed rescue therapy during the observation period of MycarinG and enrolled in MG0004 instead of receiving IVIg or PLEX, the first 6 weeks of rozanolixizumab treatment in MG0004 was considered one symptom-driven cycle. Patients from MG0004 who had received ≥6 weekly doses of rozanolixizumab moved directly into the observation period of MG0007 once MG0007 had been initiated at their study center and MG0004 closed. If their symptoms worsened, they received a symptom-driven cycle at the same dose level they received in MG0004. For patients who entered MG0007 directly from MycarinG, the initial cycle in MG0007 was assessed for disease worsening to determine whether it was symptom driven or fixed, with the latter defined as a treatment cycle received if it did not follow symptom worsening. After this initial cycle, all subsequent cycles in MG0007 were symptom driven.

Dose adjustments between 7 mg/kg and 10 mg/kg were permitted after the initial cycle in MG0007, at the investigator's discretion. If needed, patients switched dose at the beginning of a treatment cycle. If patients continued to experience moderate-to-severe symptoms despite treatment, they received rescue therapy (e.g., IVIg, subcutaneous immunoglobulin, PLEX/plasmapheresis or intravenous corticosteroids at a higher dose than their previous oral dose) and were withdrawn from the study.

The COVID-19 pandemic (pandemic status announced by the World Health Organization in March 2020^15^) occurred during MycarinG and OLE trial activity. Contingency measures were put in place to ensure patient safety in response to the pandemic.

### Pooled analysis

Data were pooled across the three studies because patients who completed MycarinG were potentially followed up in MG0004 and MG0007 (tip to tail analysis). This analysis included data from the completed MycarinG study, the first 6 weeks of the completed MG0004 study and the MG0007 study up to an interim data cut-off of July 8, 2022. Data are presented for combined 7 mg/kg and 10 mg/kg rozanolixizumab groups to enable patients to be followed throughout their repeated rozanolixizumab cycles as they moved between studies, allowing for the fact that patients could switch dose. Results separated by rozanolixizumab dose group are reported as supplementary material (Supplementary table 1; Supplementary figure 1–2). Results are also presented for the pre-specified MuSK and AChR Ab+ subgroups.

Three pools were defined to assess efficacy outcomes (pools E1, E2 and E3; see Table 1). The primary pool, E1, included patients who received at least two symptom-driven cycles, in order to assess response to symptom-driven cyclical treatment, in line with the anticipated use of rozanolixizumab in clinical practice. Pool E2 included patients who had received previous rozanolixizumab treatment and were receiving or waiting for a symptom-driven treatment cycle at the time of data cut-off in MG0007 to assess the treatment-free interval between symptom-driven cycles (time to a symptom-driven cycle from the last infusion of the previous cycle to the first infusion of the subsequent cycle). The time to re-treatment for each patient was analyzed in a subset of patients (pool E3) who received at least two consecutive symptom-driven cycles without any chronic weekly treatment in MG0004 or an initial fixed treatment cycle in MG0007.

**Table 1.** Safety and efficacy analysis pool definitions.

Pool and purpose	Definition	Studies included in pool	Number of patients (N)	Key outcomes assessed
**Efficacy pools**				
**E1 (primary efficacy pool):** To assess response to repeated symptom-driven cyclical treatment	Patients receiving ≥2 symptom-driven treatment cycles	MycarinG rozanolixizumab treatment groups* MG0004 (first 6 weeks only for patients requiring rescue therapy in the observation period of MycarinG)* MG0007 symptom-driven cycles ^†^	127	Mean change from baseline to Day 43 in MG-ADL QMG MGC MG Symptoms PRO MG-ADL, QMG, MGC and MG Symptoms PRO responder rates at Day 43 MSE rate at any time during a cycle Median time to MG-ADL response
**E2:** To assess treatment-free intervals	Patients who had received previous rozanolixizumab treatment and were receiving or waiting for a symptom-driven treatment cycle at the time of data cut-off for MG0007^‡^	MycarinG rozanolixizumab treatment groups* MG0004 (first 6 weeks only for patients requiring rescue therapy in the observation period of MycarinG)* MG0007 symptom-driven cycles^†^	167	Treatment-free intervals
**E3:** To visualize treatment-free intervals on an individual patient level without any chronic weekly or fixed treatment between cycles^§^ (a subset of pool E1)^§^	Patients receiving ≥2 consecutive symptom-driven cycles	MycarinG rozanolixizumab groups* MG0007 symptom-driven cycles^†^	110	Treatment-free intervals for individual patients (*post hoc* analysis)
**Safety pool**				
**Primary safety pool:** To assess the safety of rozanolixizumab during and after treatment cycles	Patients receiving ≥1 rozanolixizumab treatment cycle (includes treatment period and an [up to] 8-week observation period)^||^	MycarinG rozanolixizumab treatment groups* MG0007^†^	188	Mean and median number of infusions and cycles in the first 12 months Annualized number of infusions and cycles Number of patients with TEAEs

*Counted as one symptom-driven rozanolixizumab treatment cycle. ^†^Number of rozanolixizumab treatment cycles received varied between patients. Cycles included up to data cut-off July 8, 2022. ^‡^Includes time from previous rozanolixizumab treatment to the first symptom-driven treatment cycle in MG0007 and time between subsequent symptom-driven cycles in MG0007. ^§^Excluding chronic weekly treatment in MG0004 and initial fixed cycles in MG0007. ^||^Patients in MG0004 excluded because patients in MG0004 did not enter an observation period after receiving six doses of rozanolixizumab. gMG, generalized myasthenia gravis; MG-ADL, Myasthenia Gravis Activities of Daily Living; QMG, Quantitative Myasthenia Gravis.

Safety outcomes were assessed in all rozanolixizumab-treated patients who received at least one treatment cycle followed by an (up to) 8-week follow-up period across the studies.

### Outcomes

Safety outcomes are reported in detail elsewhere.^16^ Key safety outcomes were exposure to rozanolixizumab, measured by number of infusions, number of cycles and annualized number of cycles; number of patients with TEAEs, including serious TEAEs and TEAEs leading to withdrawal. Exposure and safety outcomes were assessed in the primary safety pool (Table 1).

Key efficacy outcomes included mean change from baseline (Day 1 of cycle) to Day 43 of each cycle in MG-ADL, QMG, MGC and the MG Symptoms Patient-Reported Outcomes (PROs) scales of Muscle Weakness Fatigability, Physical Fatigue and Bulbar Muscle Weakness; number of responders on MG-ADL (≥2.0-point improvement), QMG, MGC (both ≥3.0-point improvement) and MG Symptoms PRO (Muscle Weakness Fatigability [≥16.67-point improvement], Physical Fatigue and Bulbar Muscle Weakness [≥20.0-point improvement]^17^) at Day 43 of each cycle; number of patients achieving minimal symptom expression (MSE; MG-ADL score of 0 or 1) at any time during each cycle; median time to MG-ADL response (≥2.0-point improvement) in each cycle and median treatment-free intervals. Prespecified efficacy outcomes were assessed in pool E1 except for treatment-free intervals, which were assessed in pool E2 and time to re-treatment per patient, which was assessed in pool E3. Efficacy data are shown up to six cycles (numbered as cycles 1–6).

### Statistical analysis

Data were analyzed using frequency analyses of dichotomous and categorical variables showing the number of observations and percentages; for continuous variables, the number of observations, mean, standard deviation (SD), median, minimum and maximum values are provided. Efficacy analyses for repeated treatment cycles are based on observed data in individual cycles. Patients who used rescue therapy or were withdrawn from treatment due to TEAEs in a treatment cycle were censored at that timepoint. Annualized rate of cycles or infusions was calculated using the number of cycles or infusions initiated at time of data cut-off for MG0007, divided by the time in studies (years). For baseline characteristics, the allocation of patients to treatment group was based on maximum dose received during the first treatment cycle, although patients may have switched rozanolixizumab doses within subsequent cycles. For results presented per cycle, allocation of patients to treatment groups was according to the highest dose received during each cycle. SAS version 9.4 or later was used for all data processing.

## Results

### Patients and exposure

At the time of data cut-off (July 8, 2022), 188 patients (94 [50.0%] in the 7 mg/kg group and 94 [50.0%] in the 10 mg/group for the initial cycle) had received ≥1 treatment cycle with a safety follow-up period of up to 8 weeks (Table 1). Overall, patients received a total of 3600 infusions (median 18.0 per patient; range 1–51) and a total of 678 treatment cycles (median 3.0 per patient; range 1–9) of rozanolixizumab. Total time in studies was 174.71 patient-years, with a mean (SD) time in the studies of ∼1 year (339.2 [150.2] days; median 368.0 [range 44–599] days). The mean (SD) annualized rate was 3.4 (1.8) cycles and 17.8 (10.0) infusions. Ninety-seven (51.6%) of 188 patients had >1 year of participation and had initiated a mean (SD) of 4.0 (1.7) cycles (median 4.0; range 1–7) and 21.6 (8.7) infusions (median 21.0; range 6–39) in the first year. Among these 97 patients, 92 (94.8%), 74 (76.3%), 56 (57.7%), 41 (42.3%), 22 (22.7%) and 4 (4.1%) initiated ≥2, ≥ 3, ≥ 4, ≥ 5, ≥ 6 and ≥7 cycles in the first year, respectively.

**Figure 3.**
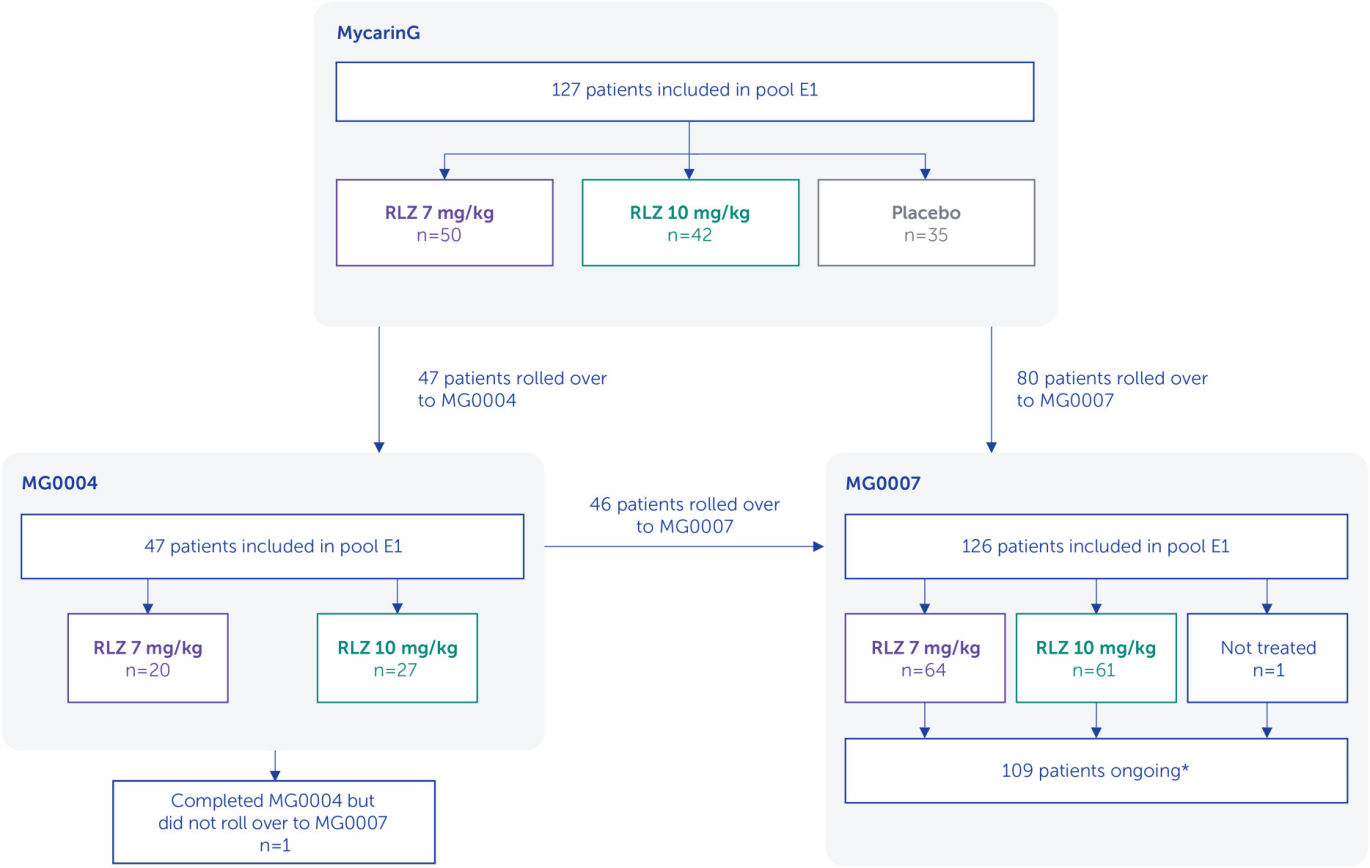
Pooled analysis patient disposition.

There were 127 patients who received ≥2 symptom-driven cycles (69 [54.3%] in the rozanolixizumab 7 mg/kg group and 58 [45.7%] in the 10 mg/kg group for the initial cycle; pool E1, Table 1). Patient disposition is shown in Figure 3. Most patients were white and enrolled in Europe or North America, and baseline characteristics were similar between treatment groups (Table 2).

Pool E1. Treatment group assignments are based on the maximum dose received in MycarinG, the maximum dose received in the symptom-driven cycle in MG0004 and the maximum dose received in the first symptom-driven cycle in MG0007; for patients who received placebo in MycarinG and rolled over to MG0004, the maximum dose received in the first 6 weeks of MG0004 was used, and for patients who received placebo and directly entered MG0007, the maximum dose received in the symptom-driven cycle of MG0007 was used. *Ongoing as of data cut-off (July 8, 2022).

**Table 2.** Baseline characteristics.

	RLZ 7 mg/kg (N = 69)*	RLZ 10 mg/kg (N = 58)*	RLZ total (N = 127)
Age, years, mean (SD)^†^	52.0 (14.3)​	48.9 (18.3)​	50.6 (16.2)​
Sex, female, n (%)​	40 (58.0)​	36 (62.1)​	76 (59.8)​
Body weight, ​n (%)​			
<50 kg​	8 (11.6)​	2 (3.4)​	10 (7.9)​
50 to <70 kg​	16 (23.2)​	24 (41.4)​	40 (31.5)​
70 to <100 kg​	32 (46.4)​	20 (34.5)​	52 (40.9)​
≥100 kg​	13 (18.8)​	12 (20.7)​	25 (19.7)​
BMI (kg/m^2^), mean (SD)	27.2 (6.7)	27.5 (6.3)	27.3 (6.5)
Geographic region, n (%)​			
North America​	19 (27.5)​	9 (15.5)​	28 (22.0)​
Europe​	40 (58.0)​	44 (75.9)​	84 (66.1)​
Asia (excl. Japan)​	2 (2.9)​	1 (1.7)​	3 (2.4)​
Japan​	8 (11.6)​	4 (6.9)​	12 (9.4)​
Race, n (%)​			
Asian​	10 (14.5)​	5 (8.6)​	15 (11.8)​
Black​	0​	1 (1.7)​	1 (0.8)​
White​	41 (59.4)​	42 (72.4)​	83 (65.4)​
Missing^‡^	18 (26.1)​	10 (17.2)​	28 (22.0)​
Age at initial MG diagnosis (years), mean (SD)	44.4 (15.7)	40.8 (19.9)	42.7 (17.7)
Duration of disease (years), median (range)	5.4 (0.4–48.9)	5.5 (0.1–35.6)	5.4 (0.1–48.9)
MGFA disease class at baseline, n (%)			
Class IIa	13 (18.8)	11 (19.0)	24 (18.9)
Class IIb	20 (29.0)	8 (13.8)	28 (22.0)
Class IIIa	19 (27.5)	24 (41.4)	43 (33.9)
Class IIIb	14 (20.3)	13 (22.4)	27 (21.3)
Class IVa	3 (4.3)	2 (3.4)	5 (3.9)
Myasthenia crisis, n (%)			
Yes	18 (26.1)	13 (22.4)	31 (24.4)
No	50 (72.5)	44 (75.9)	94 (74.0)
Missing	1 (1.4)	1 (1.7)	2 (1.6)
Baseline gMG medication, n (%)			
Corticosteroids for systemic use	41 (59.4)	38 (65.5)	79 (62.2)
Immunosuppressants	35 (50.7)	30 (51.7)	65 (51.2)
Parasympathomimetics	59 (85.5)	52 (89.7)	111 (87.4)
Thymectomy at baseline, n (%)	33 (47.8)	22 (37.9)	55 (43.3)
MG-ADL score, mean (SD)	9.1 (3.8)	8.4 (2.9)	8.8 (3.4)
QMG score, mean (SD)	16.0 (3.8)	16.0 (3.7)	16.0 (3.8)
Total IgG (g/L), mean (SD)	10.2 (2.7)	10.0 (2.7)	10.1 (2.7)
MuSK autoantibody-positive, n (%)	9 (13.0)^§^	3 (5.2)	12 (9.4)
AChR autoantibody-positive, n (%)	61 (88.4)^§^	54 (93.1)	115 (90.6)

Pool E1. *The allocation of patients to treatment group was based on the maximum dose received during the first rozanolixizumab treatment cycle, although patients may have switched rozanolixizumab doses within subsequent cycles. ^†^Missing age was calculated as year of informed consent-year of birth. ^‡^Race was not permitted to be collected from France or Canada. None of the patients were American Indian/Alaska native, native Hawaiian/other Pacific Islander or other/mixed. ^§^One patient was both AChR and MuSK antibody positive. AChR, acetylcholine receptor; BMI, body mass index; gMG, generalized myasthenia gravis; IgG, immunoglobulin G; MG, myasthenia gravis; MG-ADL, Myasthenia Gravis Activities of Daily Living; MGFA, Myasthenia Gravis Foundation of America; MuSK, muscle-specific tyrosine kinase; QMG, Quantitative Myasthenia Gravis; RLZ, rozanolixizumab; SD, standard deviation.

### Efficacy

**Figure 4.**
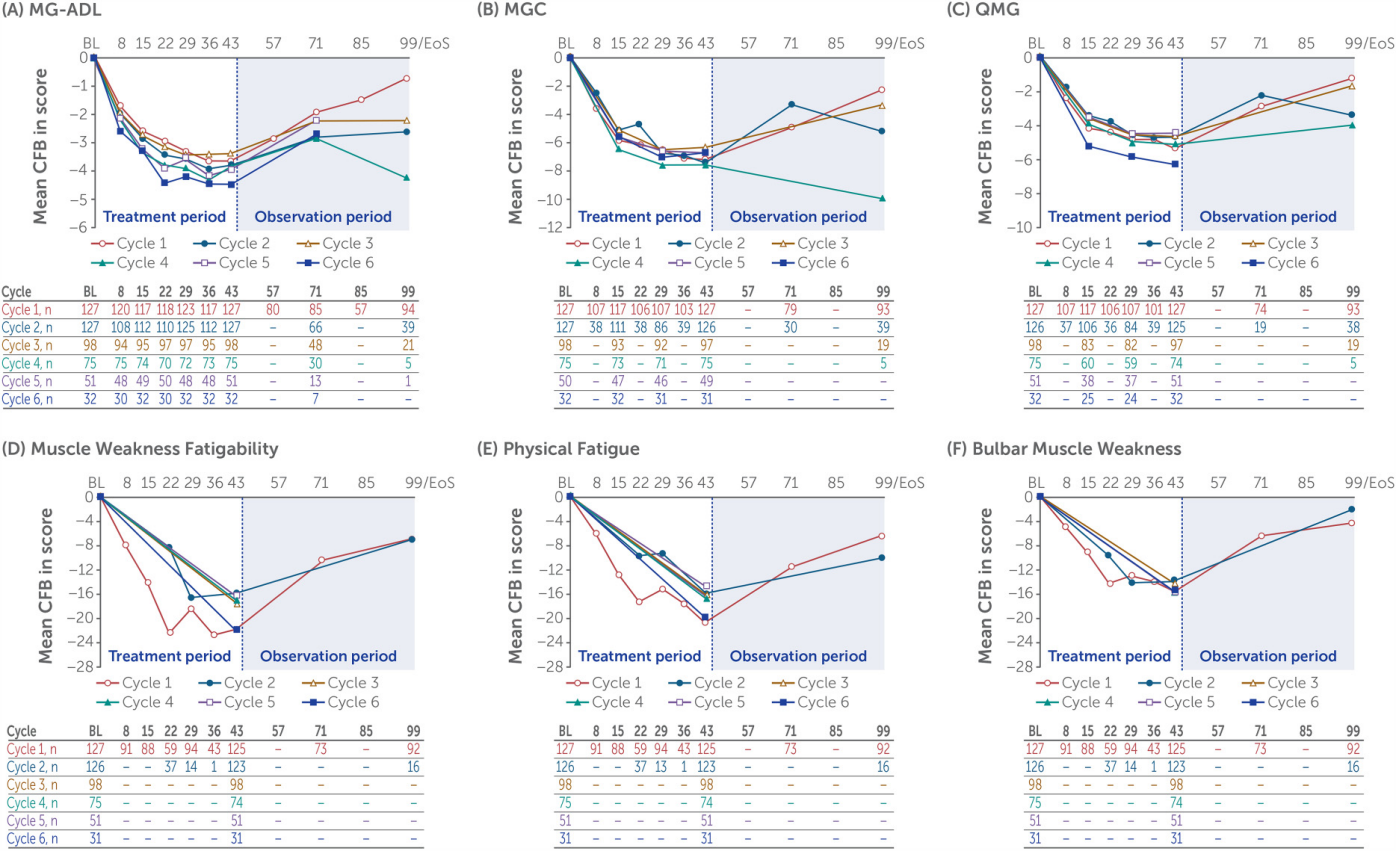
Change from baseline by treatment cycle in (A) MG-ADL, (B) MGC, (C) QMG and MG Symptoms PRO (D) Muscle Weakness Fatigability, (E) Physical Fatigue and (F) Bulbar Muscle Weakness scores for rozanolixizumab 7 mg/kg and 10 mg/kg combined.

Across repeated rozanolixizumab treatment cycles, MG-ADL scores consistently improved from the start of the cycle to the end of treatment, with rapid reductions seen as early as Day 8: mean change from baseline to Day 43 in symptom-driven cycle 1, −3.7; cycle 2, −3.9; cycle 3, −3.4; cycle 4, −3.8; cycle 5, −3.9; cycle 6, −4.5 (Figure 4). Similar reductions were observed for both dose groups separately (Supplementary figure 1). For patients with AChR Ab+ gMG (115 [90.6%] of 127), improvements in MG-ADL were similar to those observed for the overall population: mean change from baseline to Day 43 in symptom-driven cycle 1, −3.4; cycle 2, −3.7; cycle 3, −3.3; cycle 4, −3.8; cycle 5, −4.2; cycle 6, −4.7. For patients with MuSK Ab+ gMG (12 [9.4%] of 127), MG-ADL improvements were numerically higher than in the overall population for cycles 1–4 (mean change from baseline to Day 43 in symptom-driven cycle 1, −7.0; cycle 2, −5.7; cycle 3, −4.7; cycle 4, −4.2), with a lower improvement for cycle 5 (−1.3). Two patients with MuSK Ab+ gMG received cycle 6 at data cut-off; mean change from baseline was not calculated. Improvements from baseline in MGC, QMG and MG Symptoms PRO scores were observed throughout repeated cycles for the overall population (Figure 4, Supplementary figure 1) and patients with AChR and MuSK Ab+ gMG (data not shown). High MG-ADL, MGC and QMG responder rates were observed at Day 43 in symptom-driven cycle 1, with a consistent response observed over repeated cycles of treatment for the overall population (Table 3; Supplementary table 1) and patients with AChR and MuSK Ab+ gMG (data not shown).

Pool E1. BL, baseline; CFB, change from baseline; EoS, end of study; MG-ADL, Myasthenia Gravis Activities of Daily Living; MGC, Myasthenia Gravis Composite; MG Symptoms PRO, Myasthenia Gravis Symptoms Patient-Reported Outcomes; QMG, Quantitative Myasthenia Gravis.

**Table 3.** Responder outcomes at Day 43 for rozanolixizumab 7 mg/kg and 10 mg/kg combined.

	Cycle 1	Cycle 2	Cycle 3	Cycle 4	Cycle 5	Cycle 6
MG-ADL responders, n/N (%)*	94/127 (74.0)	95/127 (74.8)	63/98 (64.3)	55/75 (73.3)	40/51 (78.4)	23/32 (71.9)
MGC responders, n/N (%)^†^	93/127 (73.2)	95/127 (74.8)	68/98 (69.4)	55/75 (73.3)	35/50 (70.0)	22/31 (71.0)
QMG responders, n/N (%)^†^	87/127 (68.5)	78/125 (62.4)	63/97 (64.9)	51/74 (68.9)	30/51 (58.8)	21/32 (65.6)
MG Symptoms PRO: Muscle Weakness Fatigability responders, n/N (%)^‡§^	72/125 (57.6)	48/123 (39.0)	44/98 (44.9)	35/74 (47.3)	21/51 (41.2)	18/31 (58.1)
MG Symptoms PRO: Physical Fatigue responders, n/N (%)^§||^	55/125 (44.0)	48/123 (39.0)	31/98 (31.6)	25/74 (33.8)	20/51 (39.2)	14/31 (45.2)
MG Symptoms PRO: Bulbar Muscle Weakness responders, n/N (%)^§||^	40/125 (32.0)	37/123 (30.1)	28/98 (28.6)	27/74 (36.5)	18/51 (35.3)	10/31 (32.3)
MSE, n/N (%)^¶^	35/127 (27.6)	34/127 (26.8)	25/98 (25.5)	24/75 (32.0)	17/51 (33.3)	13/32 (40.6)

Pool E1; observed data. * ≥ 2.0-point improvement. ^†^≥3.0-point improvement. ^‡^≥16.67-point improvement. ^§^Patients with missing data at the timepoint of interest or who received rescue medication after Day 43 were treated as missing. ^||^≥20-point improvement. ^¶^MG-ADL score of 0 or 1 at any visit for each 6-week cycle and observation period. MG-ADL, Myasthenia Gravis Activities of Daily Living; MGC, Myasthenia Gravis Composite; MSE, minimal symptom expression; MG Symptoms PRO, Myasthenia Gravis Symptoms Patient-Reported Outcomes; QMG, Quantitative Myasthenia Gravis.

Some patients who did not respond initially in their first cycle achieved a response in later cycles. Of the 33 patients who were MG-ADL non-responders in the first cycle and had received ≥2 cycles, 21 (63.6%) responded in the second cycle. Median time to MG-ADL response for the combined rozanolixizumab dose group across cycles 1–6 was between 10 and 15 days.

**Figure 5.**
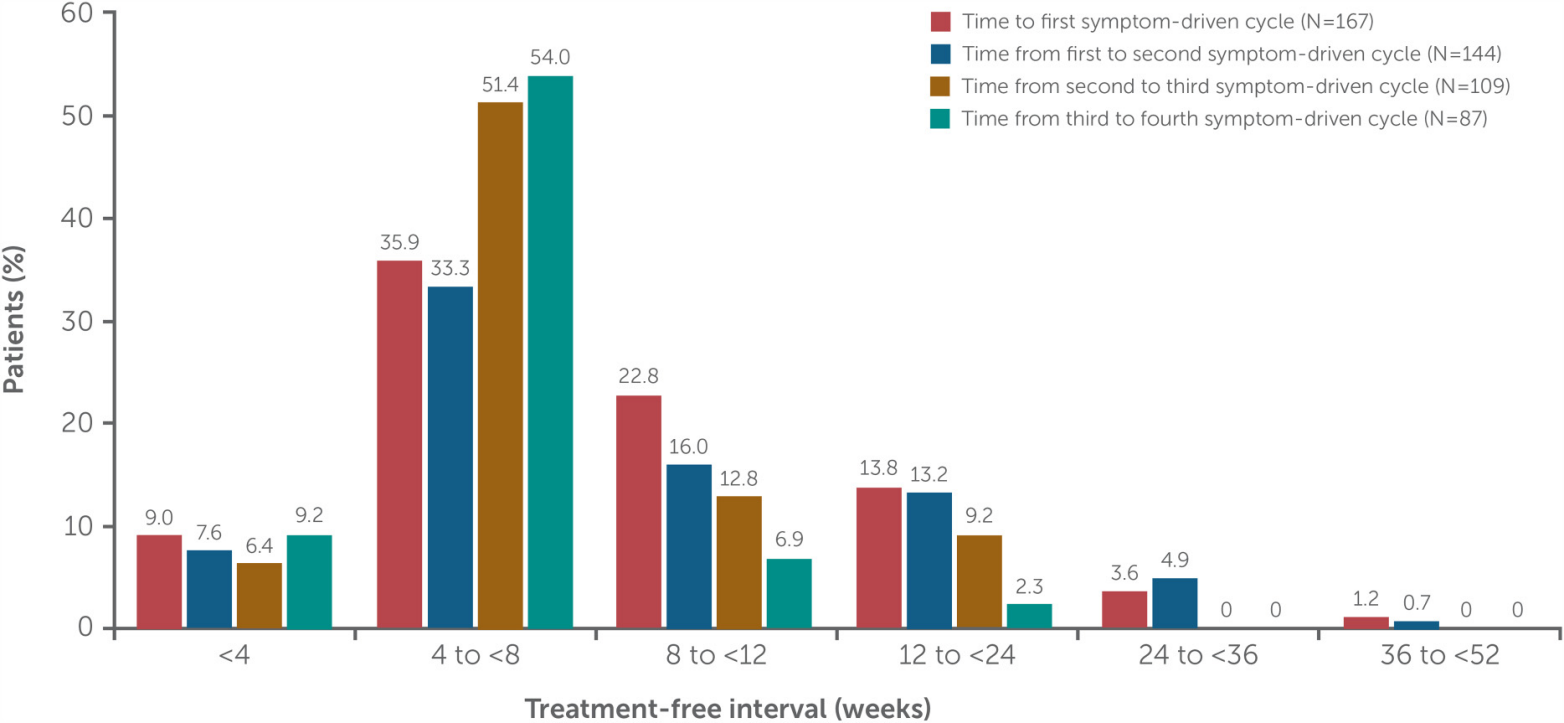
Frequency of treatment-free intervals for rozanolixizumab 7 mg/kg and 10 mg/kg combined.

The median treatment-free interval to the first symptom-driven cycle in MG0007 was 63 days (∼9 weeks) for the overall population, with a similar treatment-free interval (64 days, ∼9 weeks) between the first and second symptom-driven cycles (pool E2; Table 4). Irrespective of treatment cycle or dose, the most common treatment-free interval was between 4 and <8 weeks (Figure 5, Supplementary figure 2). From cycle to cycle, ∼10% of patients had a treatment-free interval of <4 weeks. In a *post hoc* analysis of patients who received at least two consecutive symptom-driven treatment cycles (i.e., without any chronic weekly treatment in MG0004 or initial fixed cycle in MG0007; pool E3), the number of cycles varied from two to eight cycles (pool E3; Supplementary figure 3). Treatment-free interval lengths varied between and within patients, with a more consistent treatment-free interval pattern observed for patients receiving more frequent treatment cycles.

Pool E2. Data for patients who had received rozanolixizumab treatment and had initiated the next treatment cycle based on gMG symptom worsening (N = 167). Includes up to four cycles only. Patients without a first symptom-driven cycle after rozanolixizumab treatment (time to cycle 1), second symptom-driven cycle (time between cycle 1 and cycle 2), third symptom-driven cycle (time between cycle 2 and cycle 3) or fourth symptom-driven cycle (time between cycle 3 and cycle 4) were censored at time of dropping out, data cut-off date or end of the study (MycarinG or MG0007). gMG, generalized myasthenia gravis.

**Table 4.** Median time to symptom-driven cycle for rozanolixizumab 7 mg/kg and 10 mg/kg combined by subgroup.

	Subgroups			
Time to symptom-driven cycle	Previously treated and having/waiting for first symptom-driven cycle (N = 167) Days, median (Q1–Q3)*	Had first cycle and having/waiting for second symptom-driven cycle (N = 144) Days, median (Q1–Q3)*	Had second cycle and having/waiting for third symptom-driven cycle (N = 109) Days, median (Q1–Q3)*	Had third cycle and having/waiting for fourth symptom-driven cycle (N = 87) Days, median (Q1–Q3)*
1	63 (36–105)^†^	51 (36–72)	44 (36–64)	43 (35–64)
2	-	64 (36–121)^‡^	45 (35–80)	43 (32–65)
3	-	-	43 (35–69)^§^	38 (30–62)
4	-	-	-	37 (30–55)^||^

Pool E2. Participants without a symptom-driven cycle (cycle 1) or subsequent symptom-driven cycles (cycles 2, 3 or 4) after rozanolixizumab treatment (waiting for a second, third or fourth symptom-driven cycle, respectively) are censored at time of dropping-out, data cut-off or end of MG0007); participants who had a previous cycle already censored are not included in the further subgroups. The time to a symptom-driven cycle was the time from the last infusion of the previous cycle to the first infusion of the next cycle (or date of censoring). *Kaplan-Meier estimates. ^†^23 patients were censored. ^‡^35 patients were censored. ^§^22 patients were censored. ^||^24 patients were censored.

Mean total IgG serum concentrations displayed a rapid, substantial and sustained decrease during all cycles (pool E1), with reductions seen as early as Day 8 and no evidence of loss of response in later cycles: mean change from baseline to Day 43 in symptom-driven cycle 1, −6.71 g/L, cycle 2, −5.11 g/L, cycle 3, −5.32 g/L, cycle 4, −4.64 g/L, cycle 5, −4.06 g/L, cycle 6, −4.44 g/L. AChR (cycles 1–6: −5.86, −6.08, −5.64, −6.23, −3.92, −3.56) and MuSK (cycles 1–4: −17.28, −31.69, −40.23, −39.87) autoantibody levels also decreased over time.

### Safety

Full safety results are reported separately.^16^ In the primary safety pool (N = 188), TEAEs occurred in 169 (89.9%) patients; incidence was higher in the rozanolixizumab 10 mg/kg group than in the 7 mg/kg group and did not increase with repeated cycles. The most common TEAEs were headache, diarrhea, pyrexia, nausea, COVID-19 infection, arthralgia and decreased blood IgG. The incidence of severe and serious TEAEs and of TEAEs leading to discontinuation did not increase with repeated cycles of treatment but was higher in the rozanolixizumab 10 mg/kg group than in the rozanolixizumab 7 mg/kg group.

## Discussion

This pooled analysis of Phase 3 data demonstrated that repeated 6-week treatment cycles of rozanolixizumab, at doses of 7 mg/kg or 10 mg/kg, resulted in rapid and sustained clinically meaningful improvements across all efficacy endpoints assessed. Rozanolixizumab was generally well tolerated and had an acceptable safety profile. Rozanolixizumab is a treatment option for patients with AChR Ab+ or MuSK Ab+ gMG, including those who do not exhibit adequate symptom control or cannot tolerate conventional treatments such as immunosuppressants and corticosteroids.^1,10,18^

In each treatment cycle, response to rozanolixizumab was rapid, being observed as early as Day 8 (the first timepoint at which response was assessed). This finding is similar to other targeted treatments for MG, including efgartigimod (an FcRn inhibitor)^19^ and eculizumab,^20^ ravulizumab^21^ and zilucoplan (complement inhibitors).^6^ Importantly, response rates remained high over multiple cycles, demonstrating consistent efficacy following repeated cycles. In each cycle, at least 59% of patients reached the established clinically meaningful cut-offs of both ≥2.0-point improvement for MG-ADL and ≥3.0-point improvement for MGC and QMG,^22,23^ and at least 25% of patients reached the more stringent endpoint of MSE (MG-ADL score of 0 or 1). In addition, of the patients who did not achieve response in their first cycle, almost two-thirds went on to respond in their second cycle, indicating that there could be benefit in additional cycles of treatment even for those who do not appear to respond initially.

Improvements in MG-ADL scores were observed across cycles for patients with AChR Ab+ and MuSK Ab+ gMG. MuSK Ab+ gMG can be particularly severe and difficult to treat with conventional treatments^24^; rozanolixizumab is an additional treatment option in this difficult-to-treat subpopulation.

Rapid decreases as early as Day 8 were observed in IgG levels across cycles. AChR and MuSK autoantibody levels also decreased with each cycle; however, there was significant interpatient variability in absolute levels of autoantibodies measured by the assays used, meaning that changes in patient numbers in each cycle over time may have had a significant impact on mean values across the remaining patients at later cycles. Furthermore, use of the assays for detecting AChR or MuSK autoantibody levels at baseline for longitudinal quantitative analysis was exploratory and interpretation is limited.

Consistent reductions in the novel measure MG Symptoms PRO were also demonstrated, with 29–58% of patients across cycles achieving responder cut-offs of ≥16.67 points for Muscle Weakness Fatigability and ≥20.0 points for Physical Fatigue and Bulbar Muscle Weakness.^17^ Fatigue is a symptom particularly challenging to patients as it impacts their quality of life and ability to conduct daily activities.^25,26^ The MG Symptoms PRO instrument has advantages over established instruments, such as providing greater granularity for assessment of the cardinal symptoms of MG, with more detailed assessments of muscle weakness and fatigability, including a physical fatigue domain.^27^

Rozanolixizumab demonstrated an acceptable safety profile at both 7 mg/kg and 10 mg/kg doses, consistent with the Phase 3 MycarinG study, although incidence of TEAEs was lower in the rozanolixizumab 7 mg/kg group than in the 10 mg/kg group.^14^ Incidence of TEAEs, including infections, did not increase with repeated treatment cycles. Safety results are discussed in detail elsewhere.^16^

Use of 6-week cycles of rozanolixizumab weekly dosing allows patients to tailor their treatment based on their individual MG symptom needs as assessed by their neurologist, as well as their own preferences. The interpatient variation in duration of treatment-free intervals between symptom-driven cycles confirms the need for an individualized treatment approach.

Strengths of this analysis include the long-term nature of the data, with 188.6 patient-years of exposure to rozanolixizumab, use of clinician-reported and patient-reported outcomes to ensure measurement of treatment effects relevant to both physicians and people with gMG and inclusion of MG Symptoms PRO, a newly developed instrument assessing MG severity that includes a physical fatigue domain.^27^ Measurement performance and clinically meaningful within-patient changes in the MG Symptoms PRO instrument are being validated.^25,27^ Finally, this study included patients with MuSK Ab+ gMG, providing valuable data for this historically difficult-to-treat subtype, with the proportion in this study consistent with the general gMG population.^28^

A limitation of this pooled analysis is that MG0007 is an ongoing study; therefore, data are more limited than if the study was complete. Furthermore, not all rozanolixizumab treatment cycles in the included studies were symptom driven, thus the number of cycles included in the analysis was reduced. There is also the question of whether the presence and timing of the initial fixed cycle of MG0007 affects the need for and timing of the subsequent symptom-driven cycle. In addition, the effect of the option to change the rozanolixizumab dose at the beginning of each cycle is unknown. MG0004 and MG0007 were OLE studies and no placebo group was included. Finally, there was a potential for enrichment of the rozanolixizumab-responder population because patients who did not respond to rozanolixizumab are more likely to have discontinued the study (as would be the case in clinical practice).

In conclusion, results from this pooled analysis of the Phase 3 MycarinG study and MG0004 and MG0007 OLE studies build on the positive results of the MycarinG study and demonstrate consistent rapid efficacy over repeated cycles of rozanolixizumab and an acceptable safety profile in adult patients with both AChR and MuSK Ab+ gMG. Results support neurologist-assessed symptom-driven cycles of rozanolixizumab as an individualized treatment option for adult patients with gMG.

## Supplemental Material

sj-pdf-1-jnd-10.1177_22143602241305511 - Supplemental material for Rozanolixizumab in generalized myasthenia gravis: Pooled analysis of the Phase 3 MycarinG study and two open-label extensions Supplemental material, sj-pdf-1-jnd-10.1177_22143602241305511 for Rozanolixizumab in generalized myasthenia gravis: Pooled analysis of the Phase 3 MycarinG study and two open-label extensions by Vera Bril, Artur Drużdż, Julian Grosskreutz, Ali A Habib, Renato Mantegazza, Sabrina Sacconi, Kimiaki Utsugisawa, Tuan Vu, Marion Boehnlein, Bernhard Greve, Maryam Gayfieva, Franz Woltering, Thais Tarancon, John Vissing and on behalf of the MycarinG, MG0004 and MG0007 study investigators in Journal of Neuromuscular Diseases
