# Supplementary material for: Rozanolixizumab in generalized myasthenia gravis: Pooled analysis of the Phase 3 MycarinG study and two open-label extensions
Source: J Neuromuscul Dis. 2025 Mar 4;12(2):218–30. doi: 10.1177/22143602241305511 (PMC13142871; doi:10.1177/22143602241305511)
Supplement: sj-pdf-1-jnd-10.1177_22143602241305511 - Supplemental material for Rozanolixizumab in generalized myasthenia gravis: Pooled analysis of the Phase 3 MycarinG study and two open-label extensions [file sj-pdf-1-jnd-10.1177_22143602241305511.pdf]

Rozanolixizumab in generalized myasthenia gravis: Pooled analysis of the Phase 3 MycarinG study and two open-label extensions

SUPPLEMENTARY MATERIAL

Supplementary table 1. Responder outcomes at Day 43 for rozanolixizumab 7 mg/kg and 10 mg/kg

	Cycle 1		Cycle 2		Cycle 3		Cycle 4		Cycle 5		Cycle 6	
	RLZ 7 mg/kg	RLZ 10 mg/kg	RLZ 7 mg/kg	RLZ 10 mg/kg	RLZ 7 mg/kg	RLZ 10 mg/kg	RLZ 7 mg/kg	RLZ 10 mg/kg	RLZ 7 mg/kg	RLZ 10 mg/kg	RLZ 7 mg/kg	RLZ 10 mg/kg
MG-ADL responders, n/N (%)*	52/69 (75.4)	42/58 (72.4)	49/62 (79.0)	46/65 (70.8)	28/40 (70.0)	35/58 (60.3)	23/32 (71.9)	32/43 (74.4)	17/19 (89.5)	23/32 (71.9)	9/13 (69.2)	14/19 (73.7)
MGC responders, n/N (%)[†]	48/69 (69.6)	45/58 (77.6)	45/62 (72.6)	50/65 (76.9)	29/40 (72.5)	39/58 (67.2)	25/32 (78.1)	30/43 (69.8)	14/19 (73.7)	21/31 (67.7)	8/13 (61.5)	14/18 (77.8)
QMG responders, n/N (%)[†]	43/69 (62.3)	44/58 (75.9)	33/61 (54.1)	45/64 (70.3)	29/40 (72.5)	34/57 (59.6)	22/31 (71.0)	29/43 (67.4)	10/19 (52.6)	20/32 (62.5)	9/13 (69.2)	12/19 (63.2)
MG Symptoms PRO Muscle Weakness Fatigability responders, n/N (%)[‡§]	37/69 (53.6)	35/56 (62.5)	20/61 (32.8)	28/62 (45.2)	19/40 (47.5)	25/58 (43.1)	16/31 (51.6)	19/43 (44.2)	8/19 (42.1)	13/32 (40.6)	7/12 (58.3)	11/19 (57.9)
MG Symptoms PRO Physical Fatigue responders, n/N (%)[§II]	24/69 (34.8)	31/56 (55.4)	22/61 (36.1)	26/62 (41.9)	12/40 (30.0)	19/58 (32.8)	12/31 (38.7)	13/43 (30.2)	8/19 (42.1)	12/32 (37.5)	6/12 (50.0)	8/19 (42.1)

MG Symptoms PRO Bulbar Muscle Weakness responders, n/N (%)[§‖]	22/69 (31.9)	18/56 (32.1)	16/61 (26.2)	21/62 (33.9)	14/40 (35.0)	14/58 (24.1)	12/31 (38.7)	15/43 (34.9)	5/19 (26.3)	13/32 (40.6)	5/12 (41.7)	5/19 (26.3)
MSE, n/N (%)[¶]	18/69 (26.1)	17/58 (29.3)	20/62 (32.3)	14/65 (21.5)	13/40 (32.5)	12/58 (20.7)	13/32 (40.6)	11/43 (25.6)	10/19 (52.6)	7/32 (21.9)	5/13 (38.5)	8/19 (42.1)

Pool E1; observed data. *≥2.0-point improvement. [†]≥3.0-point improvement. [‡]≥16.67-point improvement. [§]Patients with missing data at the timepoint of interest or who received rescue medication after Day 43 were treated as missing. [‖]≥20-point improvement. [¶]MG-ADL score of 0 or 1 at any visit for each 6-week cycle and observation period. MG-ADL, Myasthenia Gravis Activities of Daily Living; MGC, Myasthenia Gravis Composite; MSE, minimal symptom expression; MG Symptoms PRO, Myasthenia Gravis Symptoms Patient-Reported Outcomes; QMG, Quantitative Myasthenia Gravis; RLZ, rozanolixizumab.

Supplementary figure 1. Change from baseline by treatment cycle in (A) MG-ADL, (B) MGC, (C) QMG and MG Symptoms PRO (D) Muscle Weakness Fatigability, (E) Physical Fatigue and (F) Bulbar Muscle Weakness scores for rozanolixizumab 7 mg/kg and 10 mg/kg

(A) MG-ADL

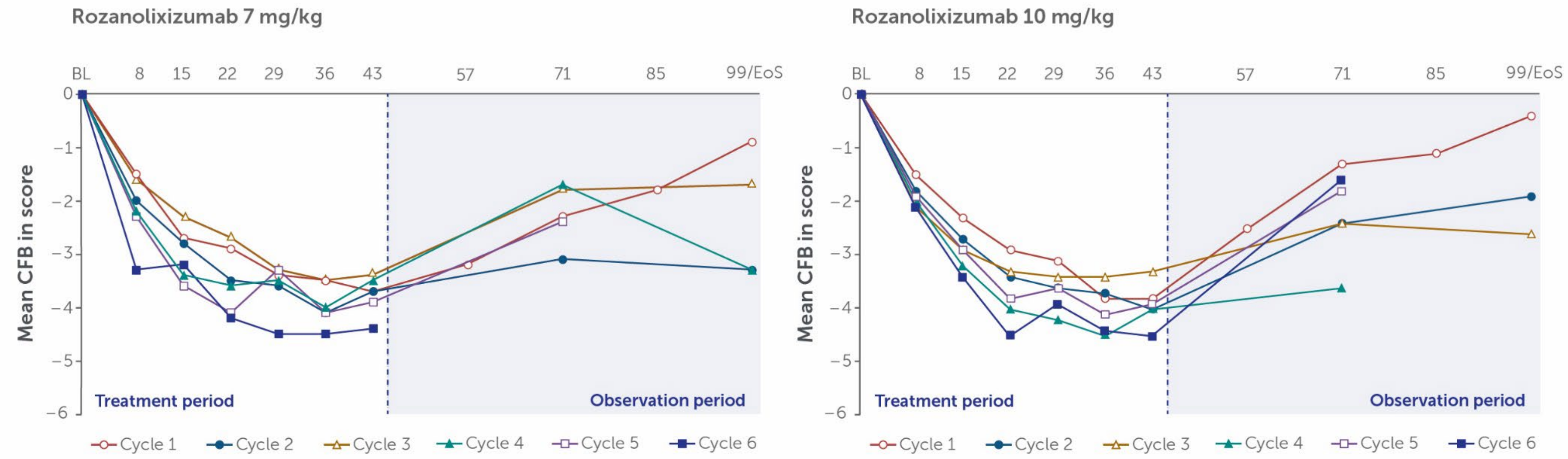

Rozanolixizumab 7 mg/kg:

Cycle	BL	8	15	22	29	36	43	57	71	85	99
Cycle 1, n	69	66	65	64	68	66	69	42	47	31	53
Cycle 2, n	62	55	56	55	61	57	62	–	37	–	20
Cycle 3, n	40	38	38	39	40	40	40	–	21	–	10
Cycle 4, n	32	32	32	32	31	31	32	–	12	–	3
Cycle 5, n	19	19	18	19	17	19	19	–	8	–	1
Cycle 6, n	13	12	13	13	13	13	13	–	2	–	–

Rozanolixizumab 10 mg/kg:

Cycle	BL	8	15	22	29	36	43	57	71	85	99
Cycle 1, n	58	54	52	54	55	51	58	38	38	26	41
Cycle 2, n	65	53	56	55	64	55	65	–	29	–	19
Cycle 3, n	58	56	57	58	57	55	58	–	27	–	11
Cycle 4, n	43	43	42	38	41	42	43	–	18	–	2
Cycle 5, n	32	29	31	31	31	29	32	–	5	–	–
Cycle 6, n	19	18	19	17	19	19	19	–	5	–	–

(B) MGC

Rozanolixizumab 7 mg/kg

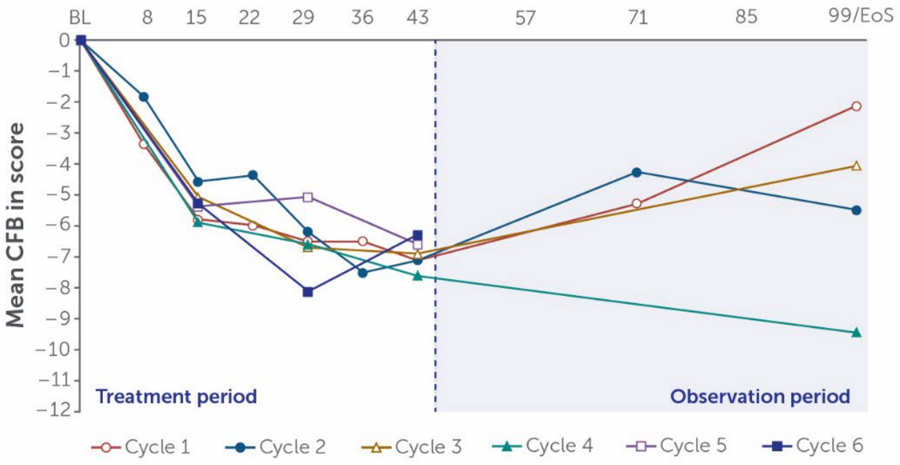

Rozanolixizumab 10 mg/kg

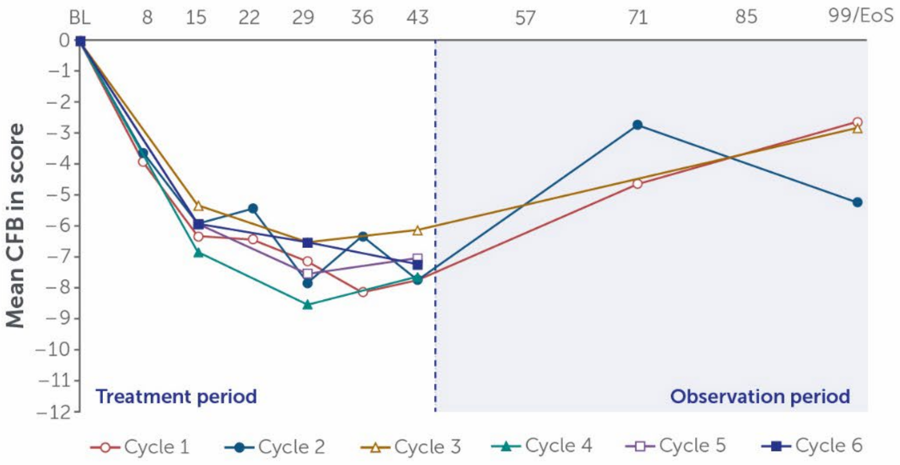

Cycle	BL	8	15	22	29	36	43	57	71	85	99
Cycle 1, n	69	58	65	57	59	58	69	–	43	–	52
Cycle 2, n	62	20	55	19	41	21	61	–	15	–	20
Cycle 3, n	40	–	37	–	40	–	40	–	–	–	9
Cycle 4, n	32	–	32	–	31	–	32	–	–	–	3
Cycle 5, n	19	–	18	–	16	–	19	–	–	–	–
Cycle 6, n	13	–	13	–	12	–	13	–	–	–	–

BL	8	15	22	29	36	43	57	71	85	99
58	49	52	49	48	45	58	–	36	–	41
65	18	56	19	45	18	65	–	15	–	19
58	–	56	–	52	–	57	–	–	–	10
43	–	41	–	40	–	43	–	–	–	2
31	–	29	–	30	–	30	–	–	–	–
19	–	19	–	19	–	18	–	–	–	–

(C) QMG

Rozanolixizumab 7 mg/kg

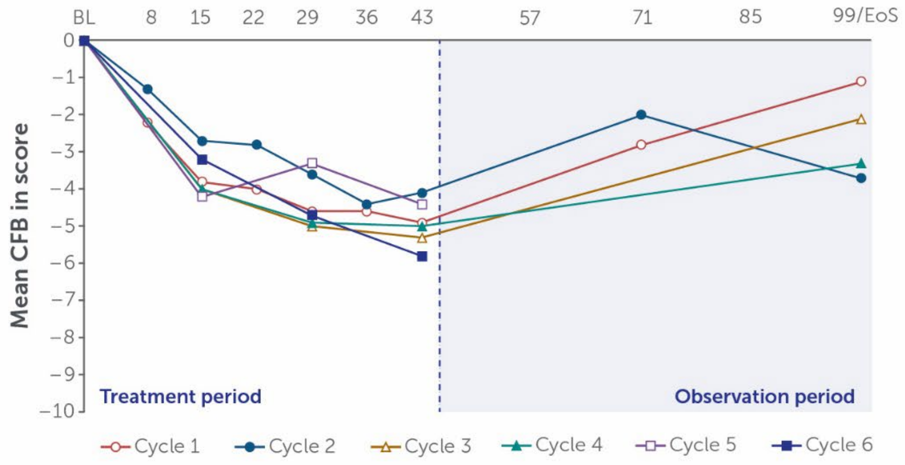

Rozanolixizumab 10 mg/kg

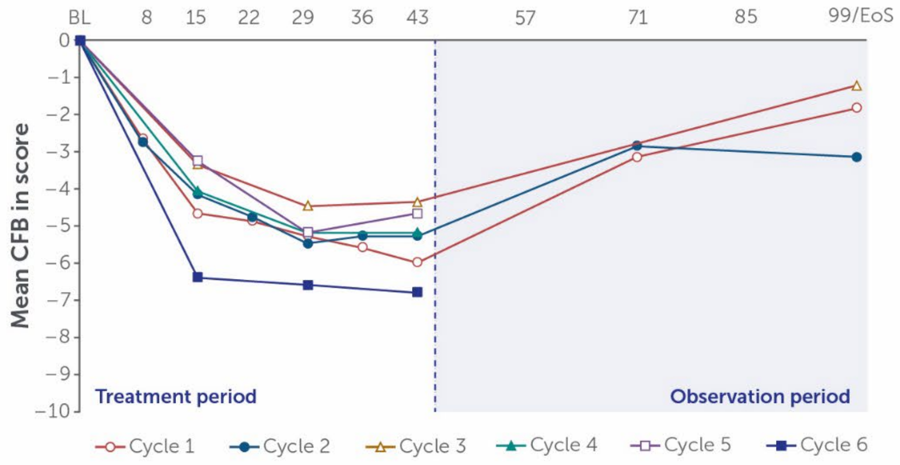

Cycle	BL	8	15	22	29	36	43	57	71	85	99
Cycle 1, n	69	58	65	57	59	58	69	–	40	–	52
Cycle 2, n	61	19	52	17	40	21	61	–	11	–	19
Cycle 3, n	40	–	31	–	33	–	40	–	–	–	9
Cycle 4, n	32	–	22	–	22	–	31	–	–	–	3
Cycle 5, n	19	–	12	–	11	–	19	–	–	–	–
Cycle 6, n	13	–	9	–	8	–	13	–	–	–	–

Cycle	BL	8	15	22	29	36	43	57	71	85	99
Cycle 1, n	58	49	52	49	48	43	58	–	34	–	41
Cycle 2, n	65	18	54	19	44	18	64	–	8	–	19
Cycle 3, n	58	–	52	–	49	–	57	–	–	–	10
Cycle 4, n	43	–	38	–	37	–	43	–	–	–	2
Cycle 5, n	32	–	26	–	26	–	32	–	–	–	–
Cycle 6, n	19	–	16	–	16	–	19	–	–	–	–

(D) Muscle Weakness Fatigability

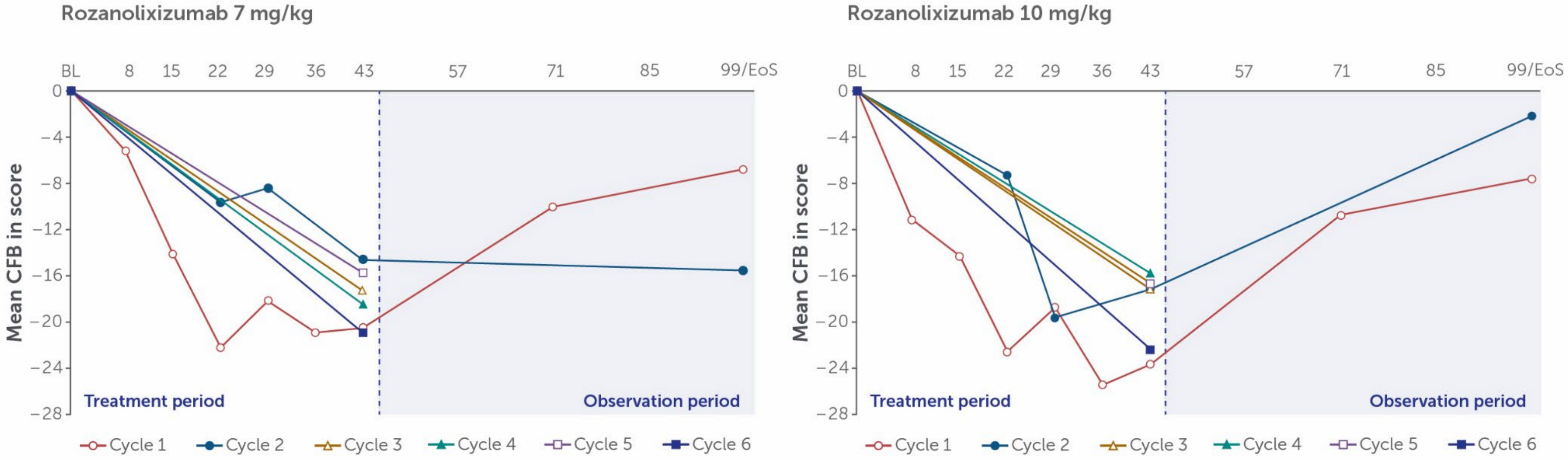

Rozanolixizumab 7 mg/kg

Cycle	BL	8	15	22	29	36	43	57	71	85	99
Cycle 1, n	69	50	49	32	51	25	69	–	39	–	51
Cycle 2, n	62	–	–	19	4	1	61	–	–	–	6
Cycle 3, n	40	–	–	–	–	–	40	–	–	–	–
Cycle 4, n	32	–	–	–	–	–	31	–	–	–	–
Cycle 5, n	19	–	–	–	–	–	19	–	–	–	–
Cycle 6, n	12	–	–	–	–	–	12	–	–	–	–

Rozanolixizumab 10 mg/kg

Cycle	BL	8	15	22	29	36	43	57	71	85	99
Cycle 1, n	58	41	39	27	43	18	56	–	34	–	41
Cycle 2, n	64	–	–	18	10	–	62	–	–	–	10
Cycle 3, n	58	–	–	–	–	–	58	–	–	–	–
Cycle 4, n	43	–	–	–	–	–	43	–	–	–	–
Cycle 5, n	32	–	–	–	–	–	32	–	–	–	–
Cycle 6, n	19	–	–	–	–	–	19	–	–	–	–

(E) Physical Fatigue

Rozanolixizumab 7 mg/kg

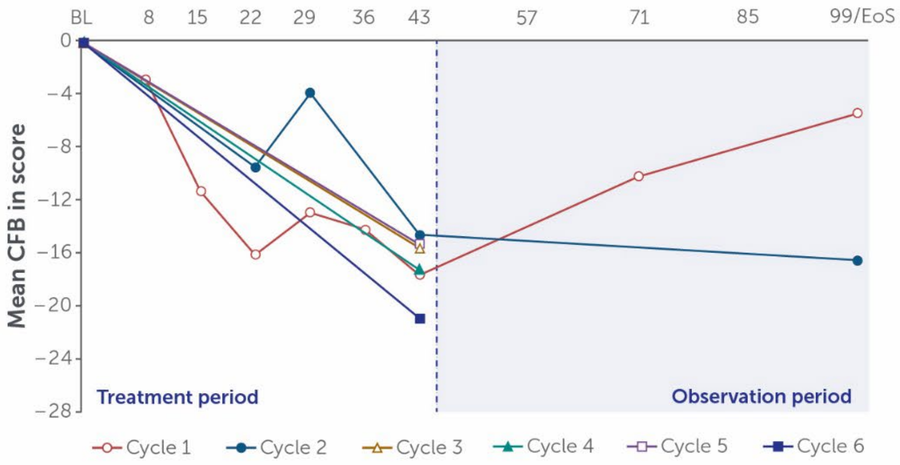

Cycle	BL	8	15	22	29	36	43	57	71	85	99
Cycle 1, n	69	50	49	32	51	25	69	–	39	–	51
Cycle 2, n	62	–	–	19	4	1	61	–	–	–	6
Cycle 3, n	40	–	–	–	–	–	40	–	–	–	–
Cycle 4, n	32	–	–	–	–	–	31	–	–	–	–
Cycle 5, n	19	–	–	–	–	–	19	–	–	–	–
Cycle 6, n	12	–	–	–	–	–	12	–	–	–	–

Rozanolixizumab 10 mg/kg

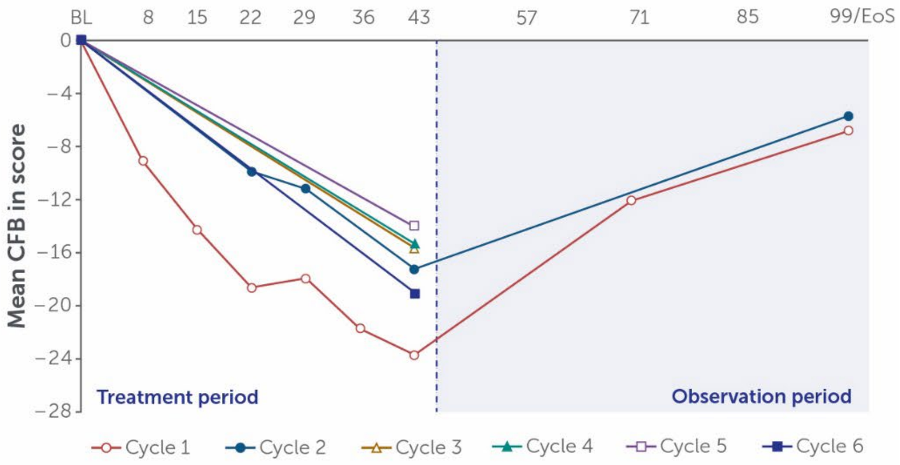

Cycle	BL	8	15	22	29	36	43	57	71	85	99
Cycle 1, n	58	41	39	27	43	18	56	–	34	–	41
Cycle 2, n	64	–	–	18	9	–	62	–	–	–	10
Cycle 3, n	58	–	–	–	–	–	58	–	–	–	–
Cycle 4, n	43	–	–	–	–	–	43	–	–	–	–
Cycle 5, n	32	–	–	–	–	–	32	–	–	–	–
Cycle 6, n	19	–	–	–	–	–	19	–	–	–	–

(F) Bulbar Muscle Weakness

Rozanolixizumab 7 mg/kg

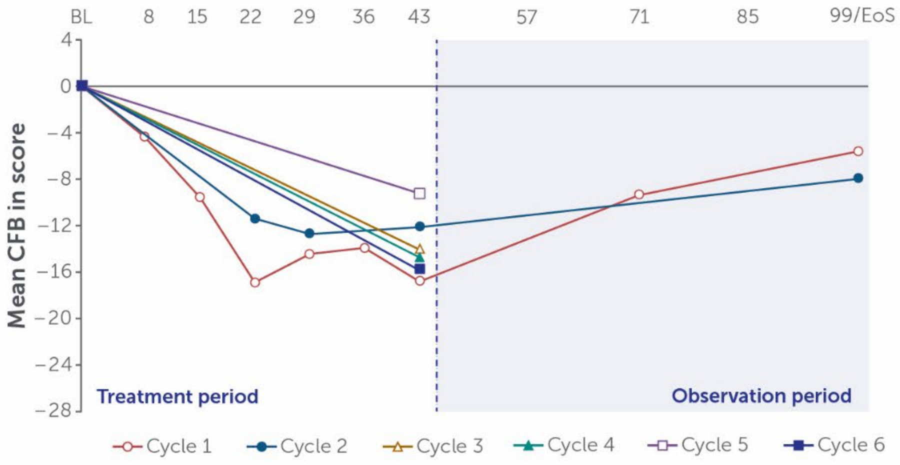

Rozanolixizumab 10 mg/kg

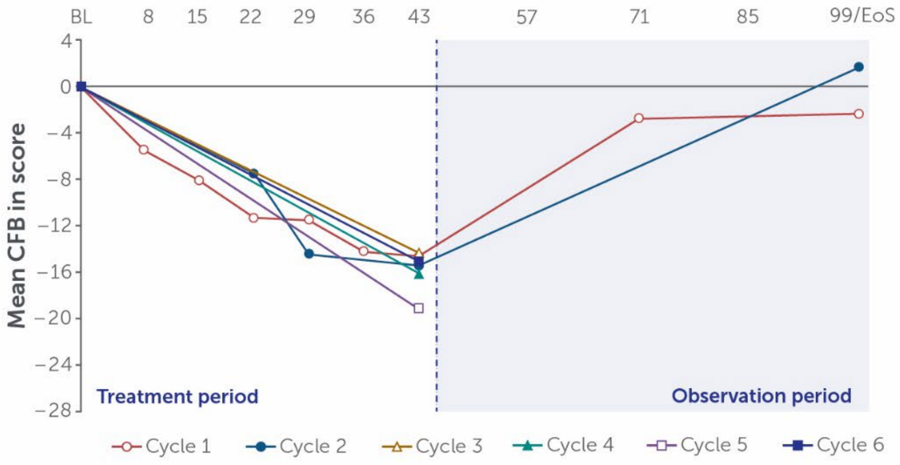

Cycle	BL	8	15	22	29	36	43	57	71	85	99
Cycle 1, n	69	50	49	32	51	25	69	–	39	–	51
Cycle 2, n	62	–	–	19	4	1	61	–	–	–	6
Cycle 3, n	40	–	–	–	–	–	40	–	–	–	–
Cycle 4, n	32	–	–	–	–	–	31	–	–	–	–
Cycle 5, n	19	–	–	–	–	–	19	–	–	–	–
Cycle 6, n	12	–	–	–	–	–	12	–	–	–	–

BL	8	15	22	29	36	43	57	71	85	99
58	41	39	27	43	18	56	–	34	–	41
64	–	–	18	10	–	62	–	–	–	10
58	–	–	–	–	–	58	–	–	–	–
43	–	–	–	–	–	43	–	–	–	–
32	–	–	–	–	–	32	–	–	–	–
19	–	–	–	–	–	19	–	–	–	–

Pool E1. BL, baseline; CFB, change from baseline; EoS, end of study; MG-ADL, Myasthenia Gravis Activities of Daily Living; MGC, Myasthenia Gravis Composite; MG Symptoms PRO, Myasthenia Gravis Symptoms Patient-Reported Outcomes; QMG, Quantitative Myasthenia Gravis.

Supplementary figure 2. Frequency of treatment-free intervals for rozanolixizumab (A) 7 mg/kg and (B) 10 mg/kg

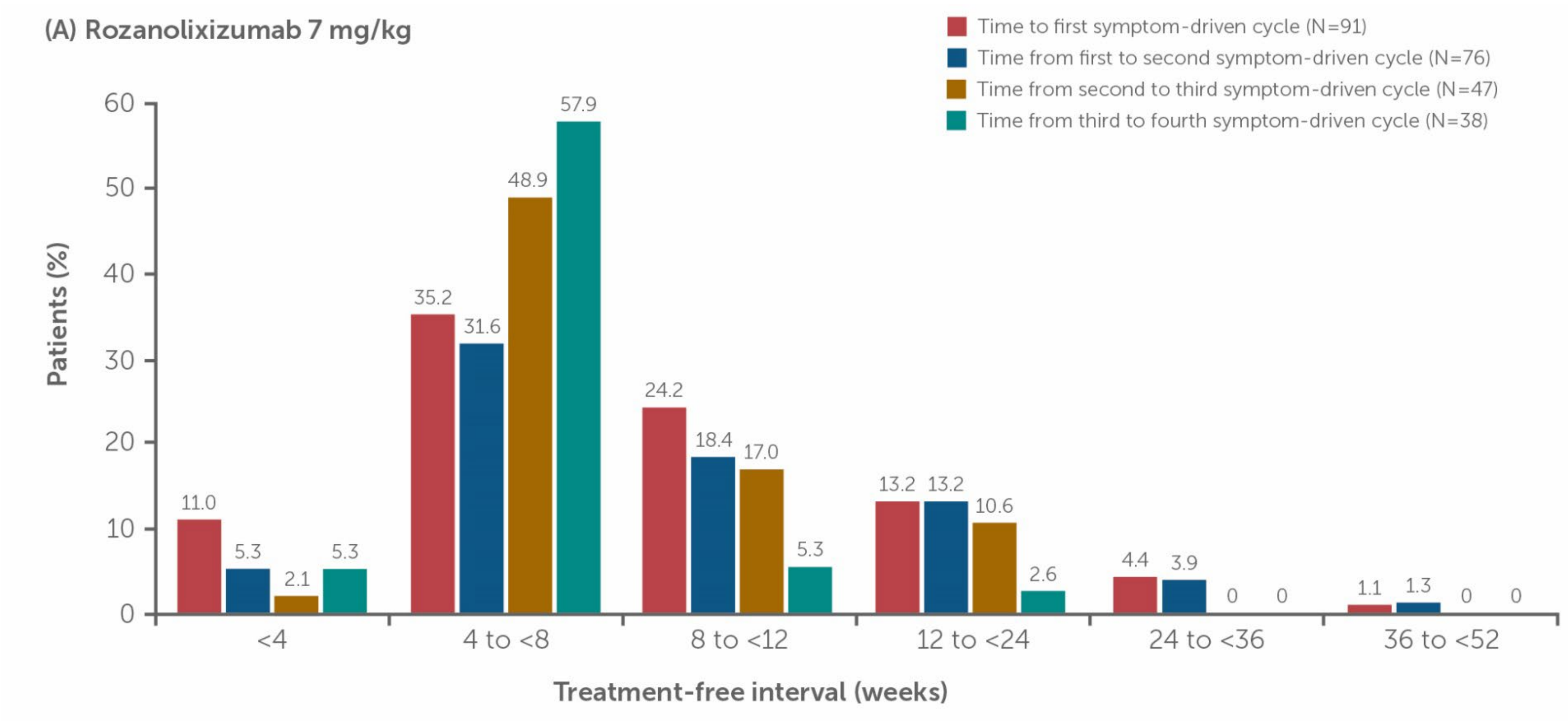

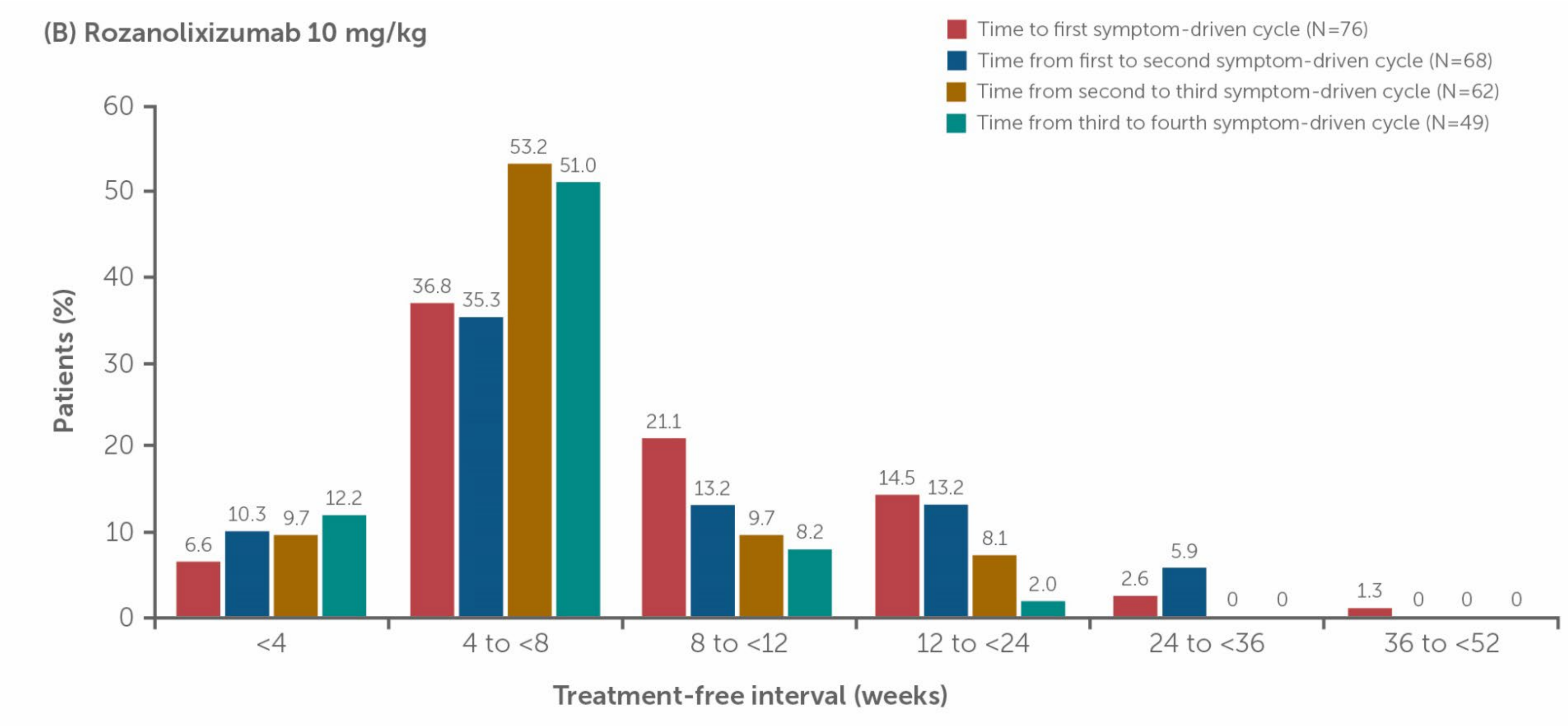

Pool E2. Data for patients who had received rozanolixizumab treatment and had initiated the next treatment cycle based on gMG symptom worsening (N=167). Includes up to four cycles only. Patients without a first symptom-driven cycle after rozanolixizumab treatment (time to cycle 1), second symptom-driven cycle (time between cycle 1 and cycle 2), third symptom-driven cycle (time between cycle 2 and cycle 3) or fourth symptom-driven cycle (time between cycle 3 and cycle 4) were censored at time of dropping out, data cut-off date or end of the study (MycarinG or MG0007). gMG, generalized myasthenia gravis.

Supplementary figure 3. Treatment-free intervals for individual patients

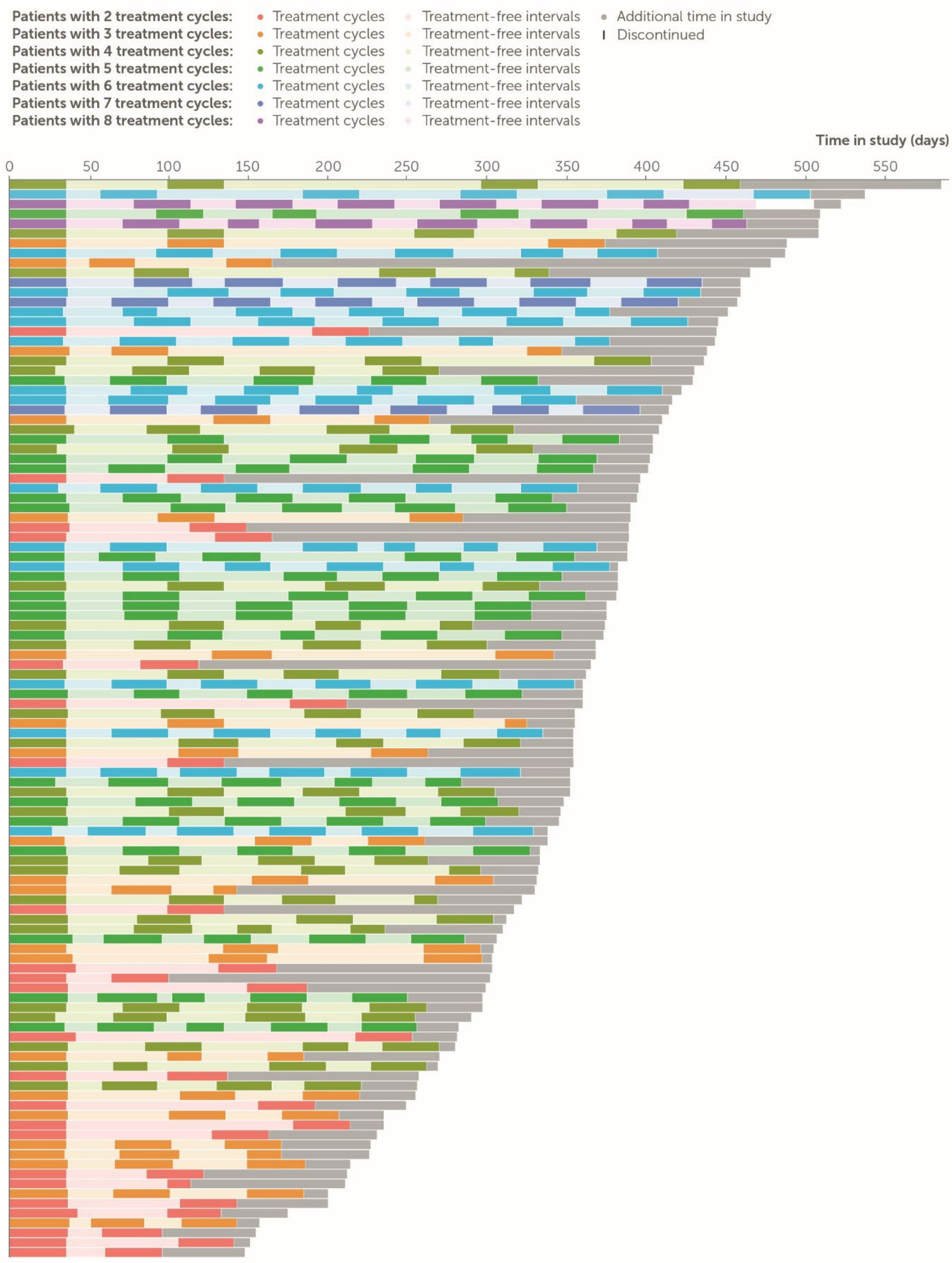

Pool E3. Each row represents an individual patient cycling through successive treatment cycles and treatment-free intervals.

Supplementary methods

MG0004 and MG0007 methodology

- Key inclusion criteria included ≥18 years of age, bodyweight ≥35 kg and male using contraception or female not pregnant/breastfeeding and using contraception or not of childbearing potential
- To be eligible to participate in MG0004 or MG0007, patients must have either completed MycarinG (including the observation period) or required rescue therapy during the observation period in MycarinG
    - Patients who did not complete the 6-week treatment period in MycarinG because they needed rescue therapy and who required initiation of a second course of rescue therapy while in the 8-week observation period could roll over to MG0004 or MG0007 if a minimum of 2 weeks had elapsed since completion of the last intravenous immunoglobulin (IVIg) or plasma exchange (PLEX) session
    - Patients who completed the treatment period of MycarinG and then opted to initiate IVIg or PLEX when they needed rescue therapy during the observation were period were not eligible to enroll in MG0004 or MG0007
- Patients who completed at least 6 visits in MG0004 were eligible to enroll in MG0007. For both studies, key exclusion criteria included meeting any mandatory withdrawal or mandatory study drug discontinuation criteria for MycarinG or discontinuing study medication in either study (with the exception in MG0004 of discontinuation due to a need for rescue treatment), a medical or psychiatric condition that would make the individual unsuitable for participation in the study, hypersensitivity to any components of the study drug or reaction after exposure to other neonatal Fc receptor inhibitors, severe weakness affecting oropharyngeal or respiratory muscles, myasthenic crisis or impending crisis at screening/baseline or an absolute neutrophil count <1500 cells/mm^3

- Additional exclusion criteria in MG0007 included meeting mandatory withdrawal or study drug discontinuation criteria in MG0004 or permanently discontinuing the study drug in MG0004
- In both studies, permitted concomitant medications were oral corticosteroids, methotrexate, mycophenolate mofetil, cyclosporin, azathioprine, cholinesterase inhibitors and tacrolimus
- In both studies, patients remained on their background generalized myasthenia gravis (gMG) medication
- In MG0007, all doses of gMG medication were maintained during each 6-week treatment period and efforts were made to maintain a stable dose during the first 8 weeks of the observation period, except for corticosteroids and acetylcholinesterase inhibitors
- In MG0007, a minimum of 4 weeks elapsing from the last dose of the previous rozanolixizumab treatment cycle before initiating a further treatment cycle was recommended
    - If a patient required treatment sooner than 4 weeks from a previous cycle, the patient's immunoglobulin G levels must have been ≥2 g/L before a new treatment cycle was initiated
- In MG0004, the primary safety outcomes were occurrence of treatment-emergent adverse events (TEAEs) and TEAEs leading to permanent discontinuation of study drug
- In MG0004, secondary efficacy outcomes were change from baseline in Myasthenia Gravis Activities of Daily Living (MG-ADL), Myasthenia Gravis Composite (MGC) and Quantitative Myasthenia Gravis (QMG) scores, and use of rescue medication
- In MG0007, the primary safety outcomes were TEAEs and TEAEs leading to withdrawal
- In MG0007, secondary efficacy outcomes were change from baseline to Day 43 within each treatment cycle (for each of the first three cycles) in MG-ADL, QMG, MGC, MG Symptoms Patient-reported Outcomes (Muscle Weakness Fatigability, Physical Fatigue and Bulbar Muscle Weakness), MG-ADL responders (≥2-point improvement), time to MG-ADL response and treatment-free intervals
